# Enhanced model for determining the number of graphene layers and their distribution from X-ray diffraction data

**DOI:** 10.3762/bjnano.6.216

**Published:** 2015-11-06

**Authors:** Beti Andonovic, Abdulakim Ademi, Anita Grozdanov, Perica Paunović, Aleksandar T Dimitrov

**Affiliations:** 1 Faculty of Technology and Metallurgy, SS Cyril and Methodius University, Rugjer Boskovik 16, 1000 Skopje, Macedonia

**Keywords:** electrochemical production, graphene, layers, X-ray diffraction

## Abstract

A model consisting of an equation that includes graphene thickness distribution is used to calculate theoretical 002 X-ray diffraction (XRD) peak intensities. An analysis was performed upon graphene samples produced by two different electrochemical procedures: electrolysis in aqueous electrolyte and electrolysis in molten salts, both using a nonstationary current regime. Herein, the model is enhanced by a partitioning of the corresponding 2θ interval, resulting in significantly improved accuracy of the results. The model curves obtained exhibit excellent fitting to the XRD intensities curves of the studied graphene samples. The employed equation parameters make it possible to calculate the *j*-layer graphene region coverage of the graphene samples, and hence the number of graphene layers. The results of the thorough analysis are in agreement with the calculated number of graphene layers from Raman spectra *C*-peak position values and indicate that the graphene samples studied are few-layered.

## Introduction

Graphene, the atom thick material that is the 2D building unit of all carbon allotropes [1], having unique and remarkable properties which are largely due to its structure, has attracted great interest in terms of fundamental studies as well as potential applications [2]. To date, several methods have been used to produce high-quality graphene sheets, such as mechanical exfoliation of graphite, chemical vapor deposition (CVD) of gases containing carbon atoms on the surface of copper films [3] and cutting open nanotubes [4]. The electrochemical approach is a proven low-cost method for a high-yield production of carbon-based nanostructures such as graphene [5–6]. Depending on the production procedure, graphene can be produced as a mixture of monolayers, bilayers and multilayers (3–10 monolayers) in form of flakes or flat sheets [7–8].

As the characterization protocol that usually follows after graphene production is an important activity, the ultimate aim of this study was to define a reliable model and thus provide a method for determining the number of graphene layers and their distribution by XRD data. To date, XRD data have been used by some authors to determine the distribution of graphene layers or their average number [9–12]. This work includes a definition and application of an enhanced model for determining the thickness distribution of graphene layers and their number by XRD data. The enhanced model was applied to graphene samples produced by two electrochemical methods: high temperature electrolysis in molten salt and electrolysis in aqueous solution, both using a nonstationary current regime. The enhancement of the model provides a great increase of the accuracy of the results, as it may be used with graphene XRD curves which are highly asymmetrical. The resulting *j*th layer region occupancies and *j*th layer coverages, for each *j* ≥ 1, allow for the calculation of the average number of layers of the graphene samples. The calculations are compared to the results obtained by other methods and are in accordance with them.

Having obtained the number of layers, together with the determined mean crystallite size *L*_a_ for the studied samples, provides a better overall picture with regard to their size and thickness. The nomenclature of the samples presented in this work is the following: graphene samples obtained by electrolysis in molten salts are denominated GMSE1, GMSE3 and GMSE4, and graphene samples produced by electrolysis in aqueous electrolyte are denominated GAE1 and GAE2.

## Results and Discussion

The XRD pattern of each of the samples was analyzed around the 002 peak, with the attention strictly focused on the line shape. Therefore, the fitting was operated in the range of 
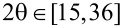
. It was done using the Laue functions model presented by Equation 1, which is further in the text addressed to as simple model [9], and by using the proposed improvement of the Laue functions model presented by Equation 2, which we name the enhanced model. The latter is recommended in general as the 002 XRD peak line shape may be extremely asymmetrical and is explained later in the text.

**Figure 1:**
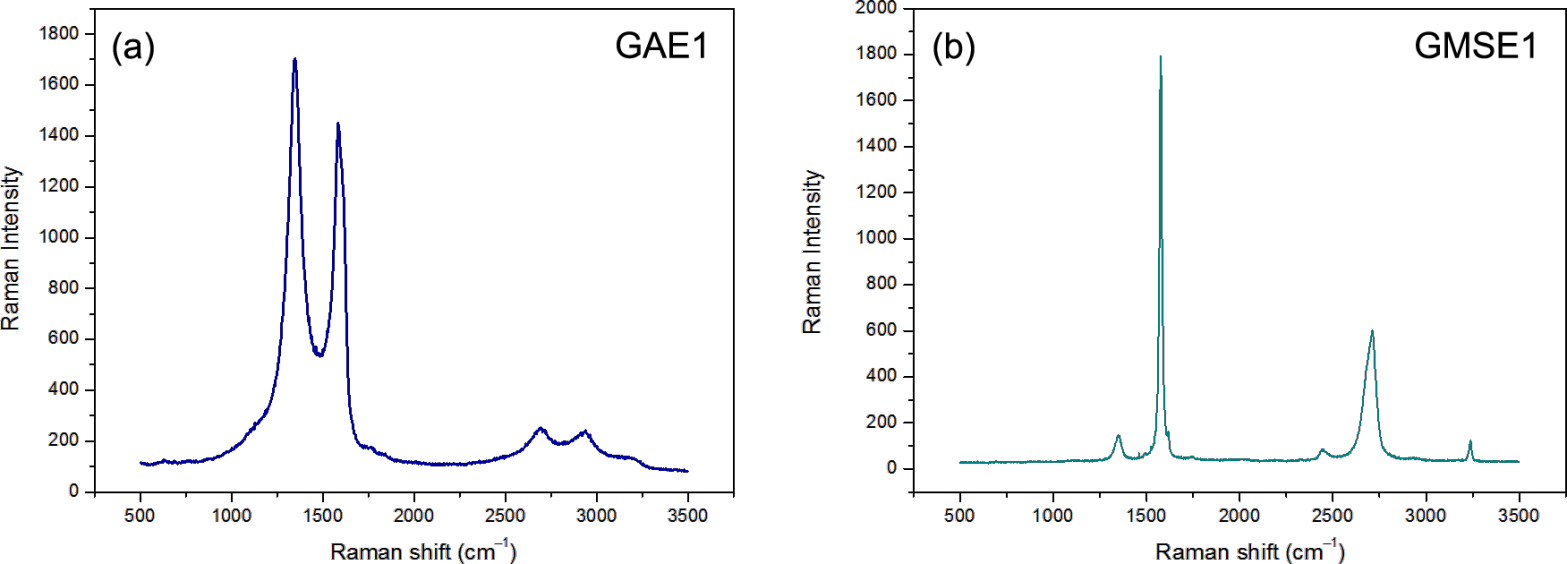
(a) Raman spectrum of GAE1; (b) Raman spectrum of GMSE1.

In Figure 1 are given the Raman spectra for two of the five presented graphene samples, GAE1 (Figure 1a), having the least symmetrical 002 XRD peak of all studied samples, and GMSE1, having an almost symmetrical 002 XRD peak, both elaborated later within this work. Raman spectra clearly show the structural order of the samples. Considering the GAE1 graphene sample, the ratio of D and G Raman intensities shows low structural order, whereas for the GMSE1 graphene sample the same ratio shows that its structure is highly ordered. This undeniably affects the symmetry of their 002 XRD peaks.

The simple model includes graphene thickness distribution and certain parameters, providing calculations of XRD intensities of the theoretical curves:

[1]
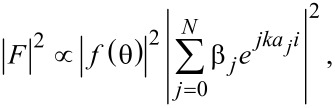


where *F* is a structure factor, *N* is the maximum number of layers, |*f*(θ)| is an atomic scattering factor which varies from 6.00 to 6.15 eV/atom with incident radiation ranging from 2 to 433 keV, *ka_j_* = (4π*d_j_* sin θ)/λ, where *d_j_* is the lattice spacing between the *j*th and the (*j* − 1)th layer, θ is an angle between the incident ray and the scattering planes, λ is the X-ray wavelength, and β*_j_*, having a value between 0 and 1, is the occupancy of *j*th graphene layer.

Hence, *B_j_* = 100β*_j_* is the occupancy of the *j*th layer in percent, and *D_j_* = *B_j_* – *B_j_*_+1_ is the *j*th-layer coverage in percent, for each *j* = 0,1,…,*N*, assuming β_0_ = 1 and β*_N_*_+1_ = 0, where *N* is the total number of layers in the studied sample, regardless of the distribution. Thus, the average number *n* of graphene layers may be calculated by the following formula:

[3]
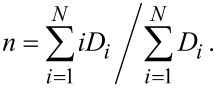


### Enhanced model for determining the number of graphene layers and their distribution by XRD data

The simple model (Equation 1) is convenient to use when the peak it is dealing with is more or less symmetrical, since this model simulates symmetrical peaks only. The approximation of the number of graphene layers therefore becomes less accurate when the 002 peak it is dealing with is highly asymmetrical. The simulation may be done with several theoretical multilayer curves, calculated from Equation 1, all being symmetrical and having an acceptable correlation coefficient to the experimental curve. The improvement to the model is done in the following way:

[2]
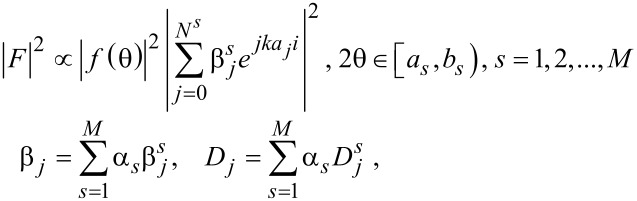


where α*_s_* is the parameter that represents the share of each simulating curve, having values between 0 and 1, and *M* is the number of simulating curves. Through Equation 2 we have defined the enhanced model. One could notice that for symmetrical peaks, there is one parameter α = 1 and thus one theoretical curve. In this case, the model in Equation 2 coincides with the model in Equation 1.

**Figure 2:**
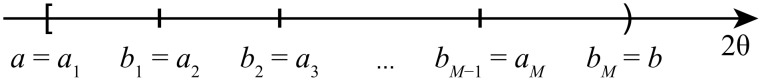
The partitioning of [a,b).

To estimate the parameters α*_s_*, we propose the partitioning of the 2θ interval [*a*,*b*) around the 002 peak. 
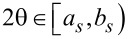
, *a*_1_ = *a*, *b_s_* = *a_s_*_+1_, *s* = 1,2,…,*s* − 1, *b_M_* = *b* and 
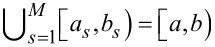
 (Figure 2). Given this, α*_s_* may be calculated through 
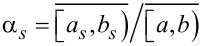
, for each *s* = 1,2,…,*M*.

Having defined the enhanced model, it is further used to analyze some graphene samples produced by electrolysis in molten salts and by electrolysis in aqueous solutions, both using reverse potential, providing considerably increased accuracy in determining graphene layers thickness distribution.

### Graphene produced by electrolysis in molten salts and the model in Equation 1

**Figure 3:**
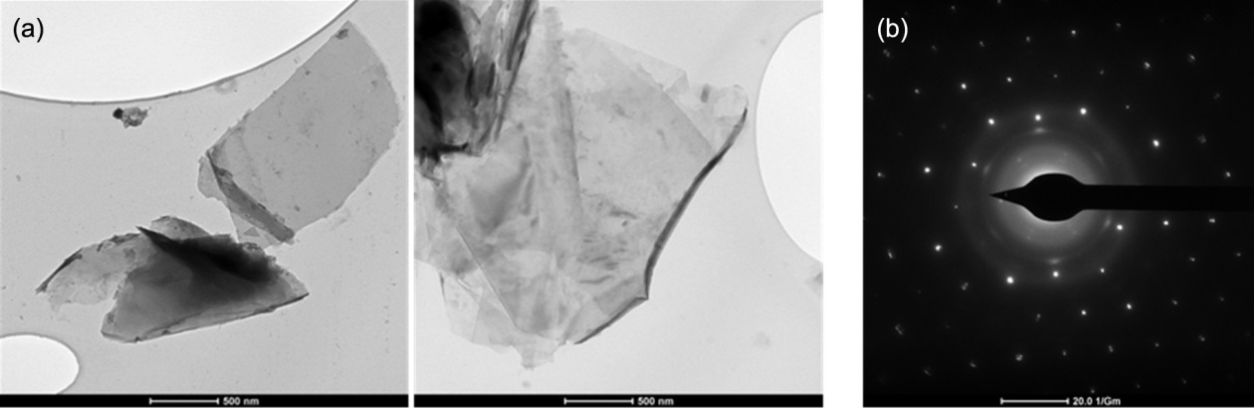
(a) TEM images of GMSE1 graphene sheets; (b) Diffraction pattern of GMSE1.

Two graphene samples produced by electrolysis in molten salts are considered and discussed herein: graphene sample GMSE1, having a symmetrical peak and graphene sample GMSE4, having an asymmetrical peak, which are different in cell potential. Typical TEM micrographs of the graphene sample GMSE1 material are shown in Figure 3a and reveal that, as usual, they are planar. The characteristic diffraction pattern of the same section is shown in Figure 3b.

**Figure 4:**
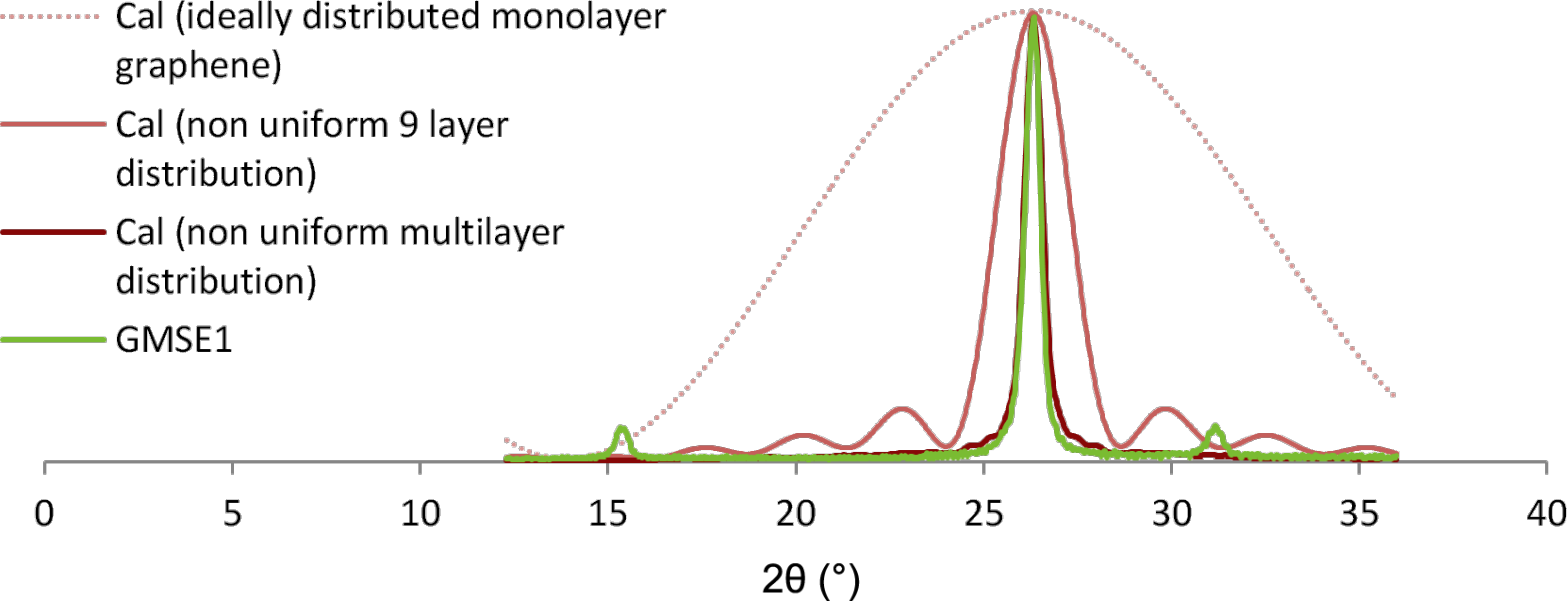
Nonuniform multilayer distribution curves for sample GMSE1 calculated from Equation 1.

Using Equation 1, XRD intensities of the curves in Figure 4 are calculated as further discussed. The three red lines are calculated curves from Equation 1 for β*_j_* ≠ 1, which suggests that the number of graphene layers has a distribution. The two light red lines are shown for comparison to the dark red fitted multilayer curve.

The wide, red dotted line in Figure 4 is the calculated curve for ideally distributed, monolayer graphene. The light red line, which is narrower than the monolayer graphene line, but broader than the green experimental curve, is the calculated curve for a nonuniform distribution of graphene layers for a 9-layered graphene sample. The dark red line is the calculated curve for a nonuniform distribution of graphene layers for multilayered graphene. This illustrates a good fit to the experimental curve of GMSE1, which is symmetrical with a correlation coefficient of ρ = 0.986. According to the associated β*_j_* parameters, the coverage of the *j*-layer graphene regions are calculated and given in Table 1.

**Table 1:** Coverage of *j*-layer GMSE1 and GMSE4 graphene regions.

GMSE1 graphene regions		GMSE4 graphene regions	

monolayer coverage	≈35%	monolayer coverage	≈62%
2–3 layer coverage	5–10%	2–4 layer coverage	5–10%
5–6 layer coverage	≈5%	5–9 layer coverage	3–5%
7–8 layer coverage	≈5%	10–15 layer coverage	≈5%
9–10 layer coverage	≈5%	15–20 layer coverage	≈2.5%
>10 layer coverage	<20%	>20 layer coverage	<25%

Apparently, the dominant structure (80% or more) is few-layered, and the average value for the number of graphene layers is calculated as *N*_GL_ = 2.87 for the dominant structure and *N*_GL_ = 5.16 for the overall structure. The calculated value of *L*_a_ for the sample GMSE1 was 1.82 nm.

**Figure 5:**
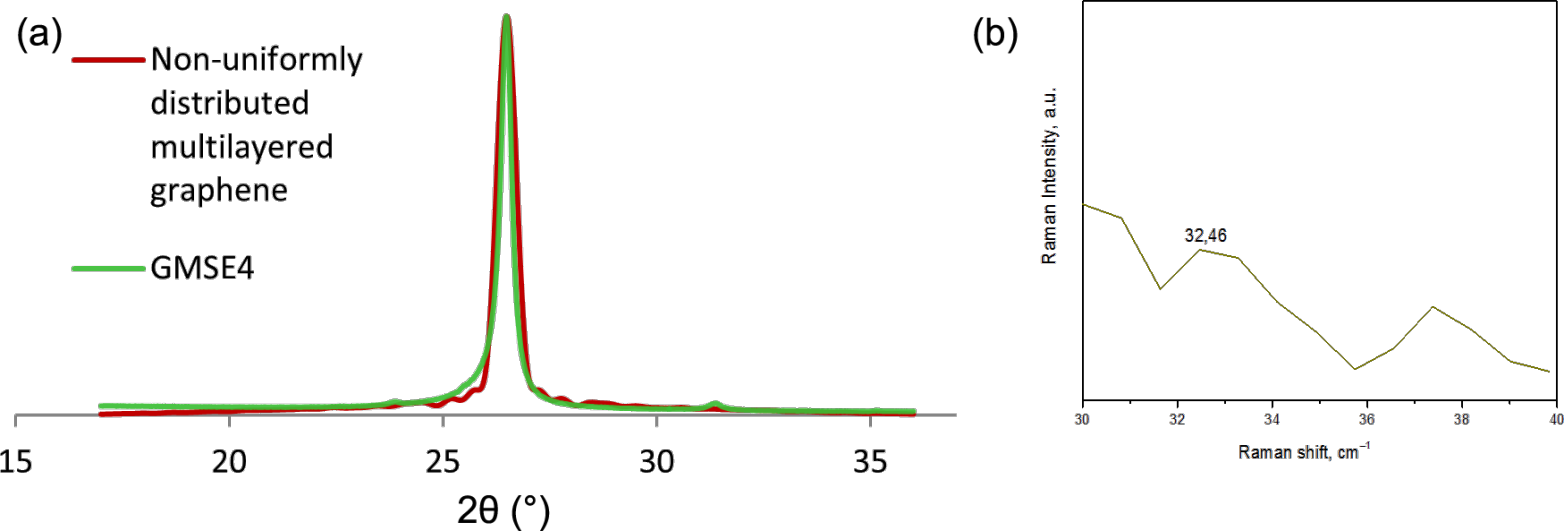
(a) Nonuniform multilayer distribution curve for sample GMSE4 calculated from Equation 1; (b) *C*-peak position in Raman spectrum for graphene sample GMSE4.

The graphene sample GMSE4 has an asymmetrical experimental 002 XRD peak, as shown in Figure 5a. The calculated curve from Equation 1 is presented in red, and the experimental curve is in green. In Figure 5b, there is a low frequency part of the Raman spectrum for sample GMSE4, given with its *C*-peak. Its position, Pos(*C*)*_N_*, is directly related to the number of graphene layers, *N*, and it varies with *N* as given by the following Equation 4 [13]:

[4]
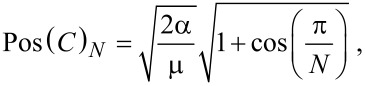


where α = 12.8 × 10^18^ N·m^−3^ is the interlayer coupling, and μ = 7.6 × 10^−27^ kg·Å^−2^ is the graphene mass per unit area.

The *j*-layer region coverages according to the analysis of the XRD 002 peak with the model in Equation 1 are given in Table 1. The average value for the number of graphene layers is calculated as *N*_GL_ = 3.1 for the dominant structure and *N*_GL_ = 9.57 for the overall structure. The calculated value of *L*_a_ for the sample GMSE4 was 2.85 nm.

According to the *C*-peak position at 32.46 cm^−1^, the number of graphene layers for sample GMSE4 is calculated as *N* = 2.13. Later within this article this graphene sample is analyzed using the enhanced model and that result is closer to the *C*-peak position method of calculation.

### Graphene produced by electrolysis in aqueous electrolyte and the model in Equation 1

**Figure 6:**
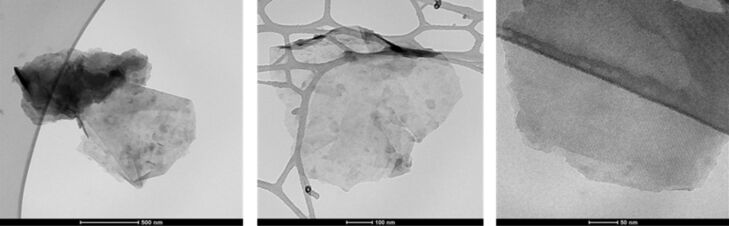
TEM images of graphene sample sheets GAE1.

Graphene samples GAE1 and GAE2 produced by electrolysis in aqueous solution from two different raw graphite materials, using nonstationary current regime, are analyzed here. Typical TEM micrographs of the graphene sample GAE1 material are shown in Figure 6. Compared to the TEM micrographs of the sample GMSE1, these indicate a higher presence of monolayer regions.

**Figure 7:**
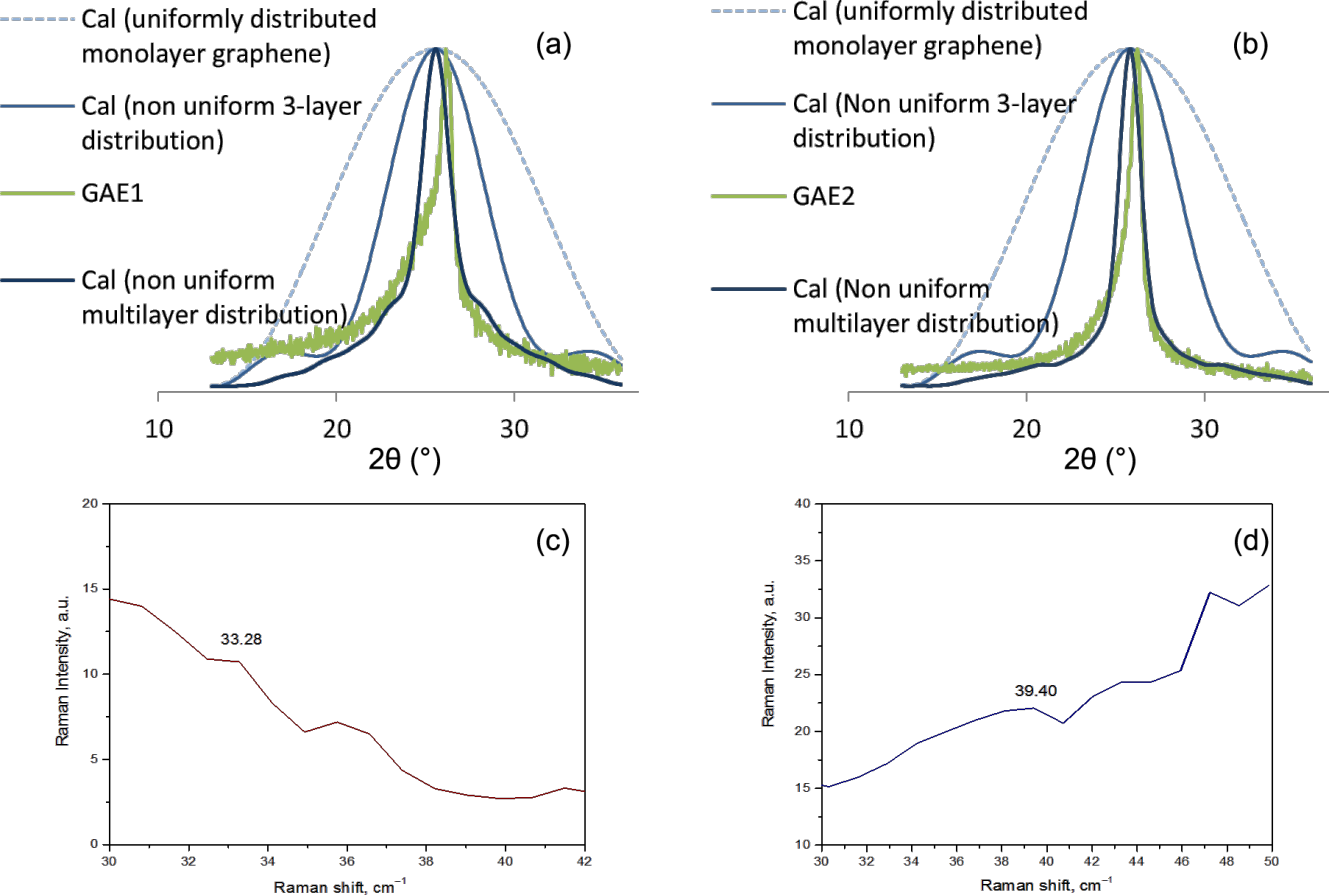
(a) Nonuniform multilayer distribution curve for sample GAE1 from Equation 1 [12]; (b) nonuniform multilayer distribution curve for sample GAE2 from Equation 1; (c) *C*-peak position in Raman spectrum for graphene sample GAE1; (d) *C*-peak position in Raman spectrum for graphene sample GAE2.

In Figure 7a and Figure 7b there is a review of the theoretical (Equation 1) nonuniform thickness curves and the experimental curves obtained for graphene samples GAE1 and GAE2. In Figure 7c and Figure 7d, there are presented the *C*-peak positions for GAE1 and GAE2, respectively.

The widest blue dotted line in Figure 7a and Figure 7b is the calculated curve for uniformly distributed monolayer graphene, the light blue line is calculated curve for a nonuniform thickness distribution of 3-layered graphene. The dark blue line is the calculated multilayer curve for a nonuniform graphene thickness distribution using the simple model of Equation 1. There is a noticeable discrepancy with the experimental curves in both Figure 7a and Figure 7b, particularly in the GAE1 spectra. Having a correlation coefficient ρ = 0.92 for sample GAE1 and ρ = 0.93 for sample GAE2, the results are acceptable and are presented in Table 2. However, as explained later in the text, using the enhanced model for analysis and sample GAE1 results in a significant improvement of the accuracy of the results.

**Table 2:** (a) Coverage of *n*-layer graphene sample GAE1 regions [12] and (b) coverage of *n*-layer graphene sample GAE2 regions.

GAE1 graphene regions		GAE2 graphene regions	

monolayer coverage	≈40%	monolayer coverage	30–35%
2 layer coverage	≈10%	3–4 layer coverage	5–10%
3–6 layer coverage	≈15%	5–6 layer coverage	5–10%
7–10 layer coverage	≈5%	7–10 layer coverage	5–10%
>10 layers coverage	<10%	>10 layer coverage	<10%

The preceding analyses show that the dominant structures of both graphene samples GAE1 and GAE2 are few-layered. The average value for the number of graphene layers of sample GAE1 is calculated as *N*_GL_ = 2.57 for the dominant graphene structure and *N*_GL_ = 4.25 for the overall graphene structure. According to the *C*-peak positions at 33.28 cm^−1^ (Figure 7c) and Equation 4, the number of graphene layers for sample GAE1 is *N* = 2.22, which is closer to the results obtained and presented later in this work by the enhanced model for the same sample. The calculated value of *L*_a_ for the sample GAE1 was 4.16 nm.

The average value for graphene layers number of sample GAE2 is calculated as *N*_GL_ = 3.53 for the dominant graphene structure and *N*_GL_ = 5.6 for the overall graphene structure. The *C*-peak positions at 39.4 cm^−1^ (Figure 7d) and Equation 4, allows for the calculation of the number of graphene layers for sample GAE2 and it is *N* = 3.46. The calculated value of *L*_a_ for the sample GAE2 was 3.93 nm.

In the following section, the alterations to the simple model, which form the enhanced model and which improve the reliability of the provided insight, the precision of the results and the fitting, are presented.

### Graphene produced by electrolysis in molten salts and the enhanced model in Equation 2

**Figure 8:**
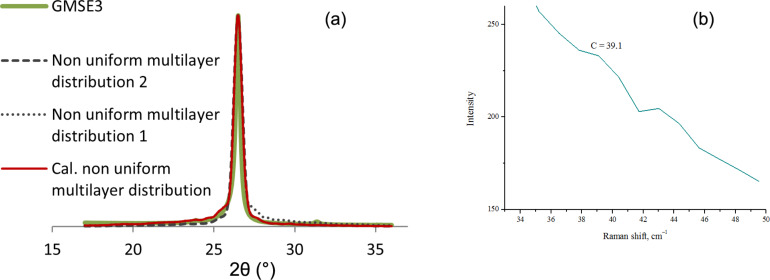
(a) Nonuniform multilayer distribution curve for sample GMSE3 calculated from Equaiton 3; (b) *C*-peak position in Raman spectrum for graphene sample GMSE3.

The graphene samples produced by electrolysis in molten salts that are considered and discussed using the enhanced model are sample GMSE3 and GMSE4. Considering GMSE3, the partitioning of the interval [17,36), is done in the following way: *I*_1_ = [17,27) and *I*_2_ = [27,36) as shown in Figure 8a. In Figure 8b, there is part of the Raman spectrum for sample GMSE3, showing its *C*-peak.

The red curve in Figure 8a exhibits a good fitting to the green experimental curve GMSE3, as its correlation coefficient is ρ = 0.96. The summarized results obtained from the calculated nonuniform multilayer distribution curve and the *j*th layer occupancy (*B_j_*) and therefore the coverages of *j*-layer graphene regions (*D_j_*) in percent for each part of the structure on the whole interval provide the analysis and results shown in Table 3.

**Table 3:** Coverage of *j*-layer GMSE3 graphene regions by the model in Equation 2.

layer distribution (in %)	*s* = 1	*s* = 2	*I* = [17,36)

α*_s_*	0.526	0.474	1
monolayer coverage	45	30	37.89
2 layer coverage	0	3	1.422
3 layer coverage	7	2	4.63
4 layer coverage	0	1	0.474
5 layer coverage	3	1	2.052
6 layer coverage	0	1	0.474
7 layer coverage	3	0	1.578
8 layer coverage	2	2	2
9 layer coverage	0	3	1.422
10 layer coverage	0	2	0.948
11 layer coverage	5	3	4.052
12 layer coverage	0	0	0
13 layer coverage	0.5	2	1.211
14 layer coverage	0.5	0	0.263
15 layer coverage	0.5	0	0.263
>15 layer coverage	≈15	≈25	≈20

The average value for number of graphene layers according to XRD 002 peak analysis according to Equation 2 is calculated as *N*_GL_ = 3.13 for the dominant graphene structure and *N*_GL_ = 8.87 for the overall graphene structure, whereas according to the *C*-peak position at 39.1 cm^−1^ and Equation 4, the number of graphene layers for sample GMSE3 is *N* = 3.3. The calculated value of *L*_a_ for the sample GMSE3 was 2.36 nm.

**Figure 9:**
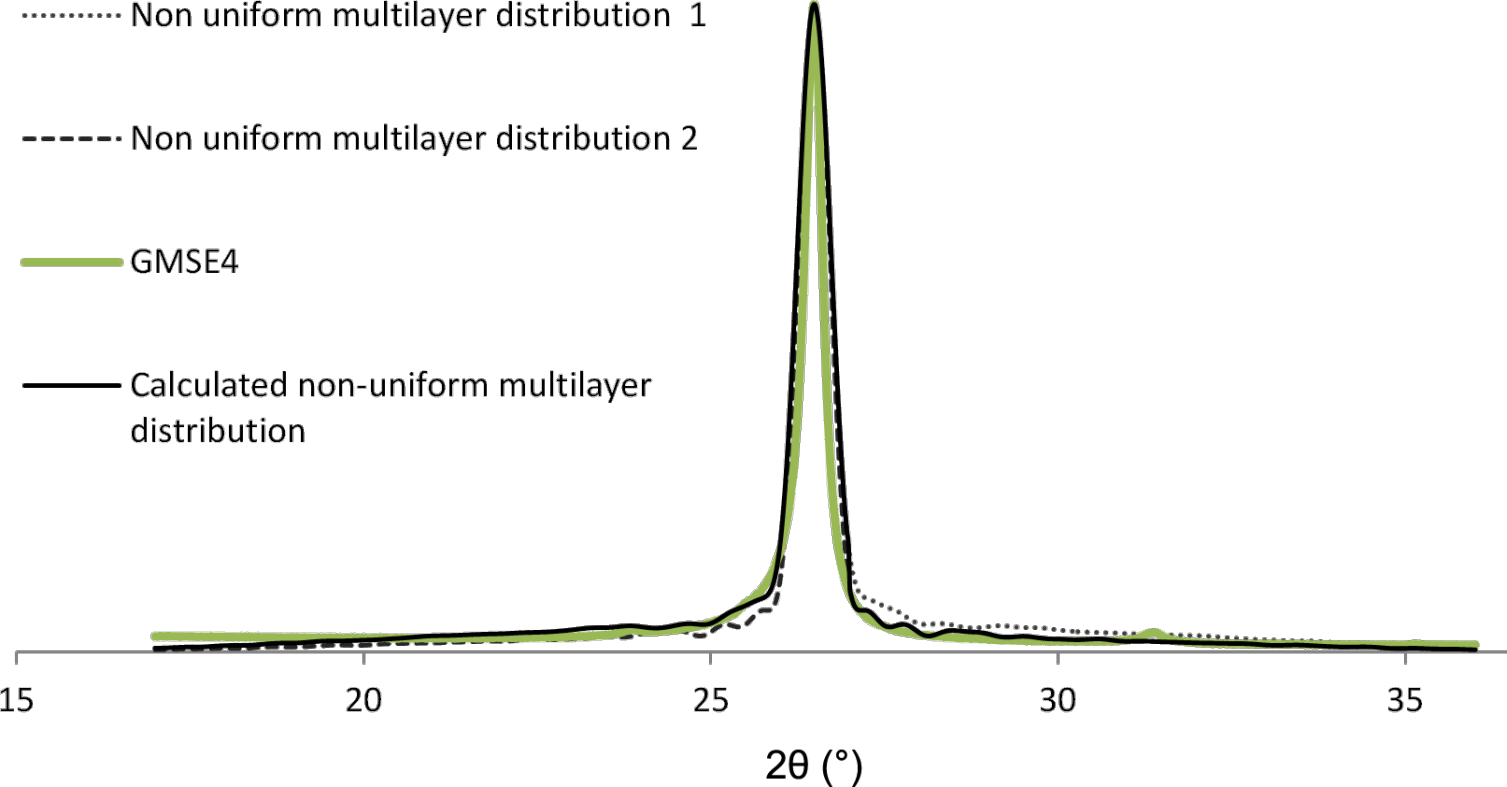
Nonuniform multilayer distribution curve for sample GMSE4 calculated from Equation 2.

Considering the sample GMSE4 and the enhanced model (Equation 2) as shown in Figure 9, we obtain the following results: *I*_1_ = [17,26.75) and *I*_2_ = [26.75,36) make the partitioning of the interval [17,36). The coverage of each *j*th layer of GMSE4 is given in Table 4.

**Table 4:** Coverage of *j*-layer GMSE4 graphene regions by the model in Equation 2.

layer distribution (in %)	*s* = 1	*s* = 2	*I* = [17,36)

α*_s_*	0.513	0.487	1
monolayer coverage	63	62	62.513
2 layer coverage	1	2	1.487
3 layer coverage	0.5	2	1.2305
4 layer coverage	0.5	1	0.7435
5 layer coverage	0.5	2	1.2305
6 layer coverage	0.5	0.2	0.3539
7 layer coverage	0.5	0.1	0.3052
8 layer coverage	0.5	0.1	0.3052
9 layer coverage	1	0.1	0.5617
10 layer coverage	1	0	0.513
11 layer coverage	3	0.5	1.7825
12 layer coverage	0	1	0.487
13 layer coverage	0.5	1	0.7435
14 layer coverage	0.5	2	1.2305
15 layer coverage	0.5	1	0.7435
>15 layer coverage	≈18	≈22	≈20

The average value for number of graphene layers according to XRD 002 peak analysis is calculated as *N*_GL_ = 2.14 for the dominant graphene structure, which is about 80% of the whole graphene sample, and *N*_GL_ = 8.68 for the overall graphene structure. One could notice that the average graphene layers number calculated using the enhanced model (Equation 2) is closer to the result obtained by the *C*-peak method than the layers number obtained by the simple model (Equation 1) calculated in the previous section.

### Graphene produced by electrolysis in aqueous electrolyte and the enhanced model in Equation 2

Graphene sample GAE1 produced by electrolysis in aqueous solution is intentionally considered for analysis again, so that the results obtained by the enhanced model could be compared to the results obtained by the previous simple model.

**Figure 10:**
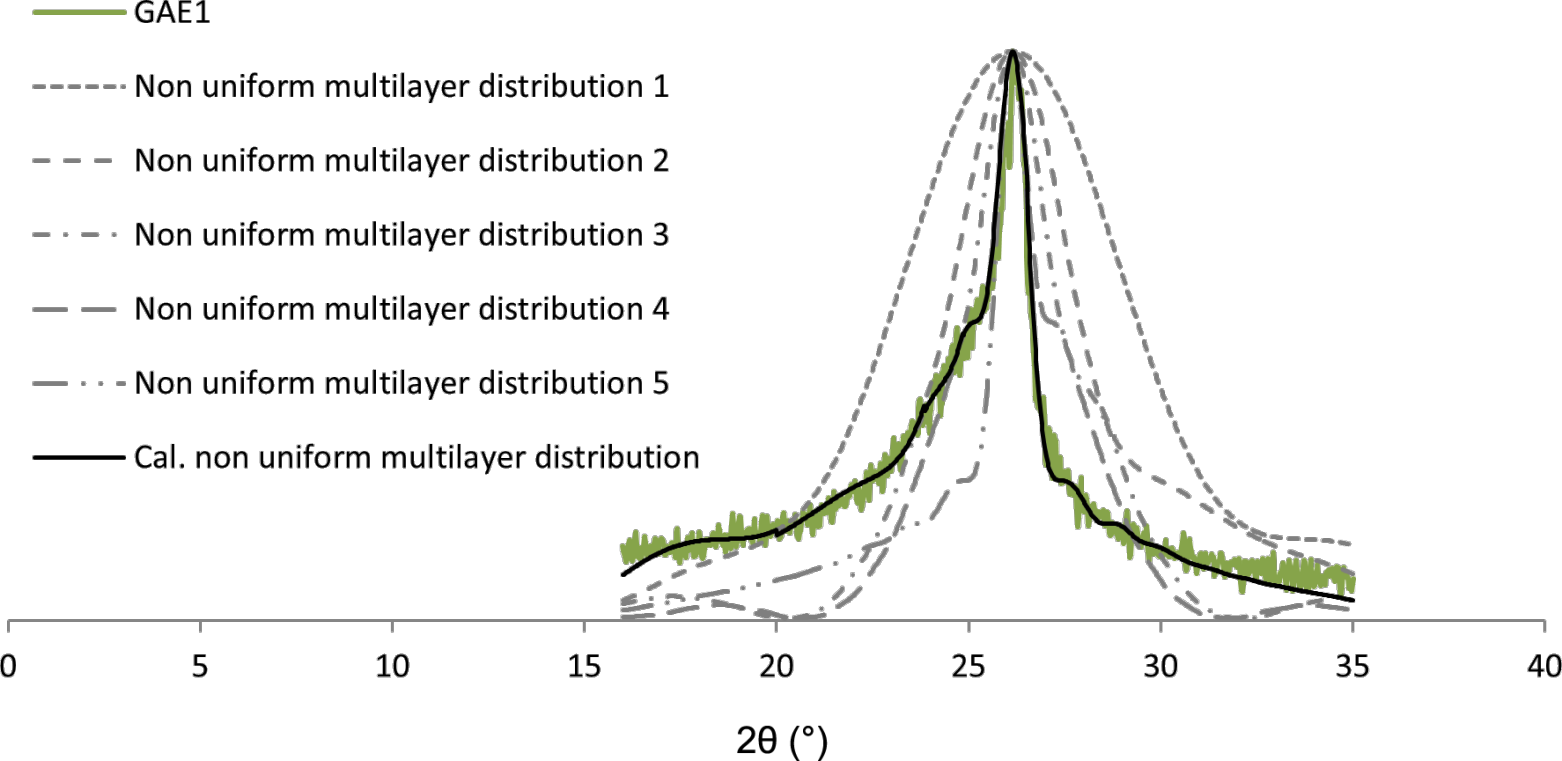
Nonuniform multilayer distribution curve for sample GAE1 calculated with Equation 2.

The interval [a,b) = [16,35) already shown in Figure 7a is now partitioned into five intervals: 
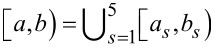
, where *I*_1_ = [16,20), *I*_2_ = [20,23.84), *I*_3_ = [23.84,24.84), *I*_4_ = [24.84,26.52) and *I*_5_ = [26.52,35). The calculations and corresponding curves obtained with the enhanced model (Equation 2) are shown in Figure 10.

The black curve in Figure 10 exhibits an excellent fitting to the green experimental curve GAE1, as its correlation coefficient is ρ = 0.99. The results obtained from the calculated nonuniform multilayer distribution curve and the *j*th layer occupancy (*B_j_*), and therefore the coverages of *j*-layer graphene regions (*D_j_*) in percent for each part of the structure over the whole interval are summarized in Table 5.

**Table 5:** Coverage of *j*-layer graphene sample GAE1 regions by the model in Equation 2.

Layer distribution (%)	*s* = 1	*s* = 2	*s* = 3	*s* = 4	*s* = 5	*I* = [16,35)

α*_s_*	0.211	0.202	0.053	0.088	0.446	1
monolayer coverage	55	58	0	0	58	49.189
2 layer coverage	0	0	0	7	2	1.508
3 layer coverage	26	6	60	30	8	16.086
4 layer coverage	0	7	13	43	2	6.779
5 layer coverage	0	2	1	0	1	0.903
6 layer coverage	0	5	0. 5	0	1	1.4825
7 layer coverage	0	3	0. 5	5	1	1.5185
8 layer coverage	0	1	0. 5	0	1	0.6745
9–30 layer coverage	0	1	7.5	5	11	<6

The graphene structure is few-layered, as nearly 95% of the overall structure consist of eight layers or less. The average value for number of graphene layers is calculated as *N*_GL_ = 2.07 for the dominant graphene structure and *N*_GL_ = 3.06 for the overall graphene structure.

## Conclusion

There are several conclusions to be drawn from the preceding analysis. The model that is used provides an additional insight into the *j*-layer occupancies and coverages of graphene samples. The enhanced model is suggested to be generally used, because graphene sheets that are subject of research may have highly asymmetrical 002 XRD peaks. Such peaks are inconvenient to be analyzed by the simple model (Equation 1), and therefore adequate alterations to the model were considered and embedded as shown in an enhanced model (Equation 2). The enhancement of the model provides a significant increase of the accuracy of the results. The results of the analyzed graphene samples, either obtained using the simple model or the enhanced model, show that these samples are few-layered. While Equation 1 already produces acceptable results when it comes to the number of layers, it is shown here that the enhanced model is in higher accordance with other methods results and therefore more accurate.

## Experimental

Graphene samples were produced by electrolysis in molten salt and aqueous solution using a nonstationary current regime (reverse potential, from 1 to 5 V, in molten salt electrolysis, and from 10 to 15 V, in aqueous solution, controlled by a molybdenum quasi-reference electrode and a calomel reference electrode). The study was done at temperatures between 400 and 600 °C in molten salt, and 25 °C in aqueous solution. It should be underlined that, during the electrolysis, the cations reduced at the electrode intercalate at the graphite surface and generate a high mechanical stress that causes exfoliation of the cathode. This phenomenon enables the electrochemical synthesis of graphene to be performed. The electrochemical route also offers the possibility for accurate control of various parameters, such as applied voltage, current density, temperature and morphology of starting material. The morphological structures of the obtained graphene samples were investigated by TEM analysis using a FEI Tecnai G2 Spirit TWIN with a LaB6 cathode. XRD spectra were recorded using a PAN-analytical X’Pert Pro diffractometer (Cu Kα radiation). Raman spectra were recorded using LABRAM ARAMIS-HORIBA JOBIN YVON system with 532 nm wavelength incident laser light, hole 250 μm, slit of 250 μm.
